# Community perceptions of health and chronic disease in South Indian rural transitional communities: a qualitative study

**DOI:** 10.3402/gha.v8.25946

**Published:** 2015-02-09

**Authors:** Arabella K. M. Hayter, Roger Jeffery, Chitra Sharma, Audrey Prost, Sanjay Kinra

**Affiliations:** 1 Department of Non-Communicable Disease Epidemiology, London School of Hygiene and Tropical Medicine, London, UK; 2 University of Edinburgh, Edinburgh, UK; 3 Public Health Foundation of India, India; 4 Institute for Global Health, University College London, London, UK

**Keywords:** chronic disease, urbanisation, community environment, qualitative, India

## Abstract

**Background:**

Chronic diseases are now the leading cause of death and disability worldwide; this epidemic has been linked to rapid economic growth and urbanisation in developing countries. Understanding how characteristics of the physical, social, and economic environment affect behaviour in the light of these changes is key to identifying successful interventions to mitigate chronic disease risk.

**Design:**

We undertook a qualitative study consisting of nine focus group discussions (FGDs) (*n=*57) in five villages in rural Andhra Pradesh, South India, to understand people's perceptions of community development and urbanisation in relation to chronic disease in rural transitional communities. Specifically, we sought to understand perceptions of change linked to diet, physical activity, and pollution (because these exposures are most relevant to chronic diseases), with the aim of defining future interventions. The transcripts were analysed thematically.

**Results:**

Participants believed their communities were currently less healthy, more polluted, less physically active, and had poorer access to nutritious food and shorter life expectancies than previously. There were contradictory perceptions of the effects of urbanisation on health within and between individuals; several of the participants felt their quality of life had been reduced.

**Conclusions:**

In the present study, residents viewed change and development within their villages as an inevitable and largely positive process but with some negative health consequences. Understanding how these changes are affecting populations in transitional rural areas and how people relate to their environment may be useful to guide community planning for health. Measures to educate and empower people to make healthy choices within their community may help reduce the spread of chronic disease risk factors in future years.

Chronic diseases, primarily cardiovascular disease (CVD), cancer, chronic obstructive pulmonary disease, and diabetes, are the leading causes of death and disability worldwide, with 80% of the deaths occurring in low- and middle-income countries (LMICs) (1). This epidemic has been linked to rapid economic development and urbanisation; exposure to the urban environment is frequently described as a determinant of health status (2–4). In India, the population is increasingly urban; at the last census in 2011, the urban population had reached 31%, with similar levels found in the state of Andhra Pradesh in South India (33.4%) (5). This is compared to 27.8 and 27.3% 10 years earlier (6). Several characteristics of the physical, social, and economic environment linked to urbanisation may influence chronic disease risk, for example air pollution, economic activity, transport infrastructure, access to healthcare and health information, shops selling processed foods and alcohol, and household ownership of consumer goods (7, 8).

According to Ecological Systems Theory, a person's behaviour cannot be explained without consideration of the environment in which they live (their ecological niche), whether it is the household, community, or city (9). The availability and accessibility of healthy lifestyle options or the restriction of unhealthy options in a person's immediate environment may have a positive impact on health outcomes (10). For example, environmental aesthetics and neighbourhood safety have been linked to increased walking and physical activity (11, 12). Similarly, the availability of healthy food options has been associated with lower levels of obesity (13), and tobacco pricing strategies and restricted availability have been shown to reduce tobacco consumption (14). The sociocultural environment may also influence behaviour negatively. For example, social norms in South Asia may prevent women from running outside because it is seen to be socially inappropriate. As rural communities undergo rapid development, it is important to understand how their environment is changing, taking into account both physical and social aspects and how these affect major chronic disease risk factors (15).

Environmental determinants of behaviour can be divided broadly into two categories – objective and perceived (7). It can be argued that people's individual and socially shaped perceptions of their environment may shape behaviour more than ‘reality’, that is, regardless of objective measures. For example, someone may perceive traffic as a threat to their safety, which prevents them from walking, when in reality the local accident rate is very low. The majority of studies looking at perceptions of the community environment on chronic disease risk focus on individual risk factors (e.g. diet or physical activity) rather than taking a more holistic approach to chronic disease risk (16–18) and many are only cross-sectional studies.

The objective of this study was to understand people's perceptions of community development and urbanisation in relation to chronic disease in rural, transitional communities in Andhra Pradesh, South India. Specifically, the intention was to understand perceptions of change linked to diet, physical activity, and pollution (because these exposures are most relevant to chronic diseases), with the aim of informing future interventions.

## Methodology

### Study design

We used qualitative methods to understand people's perceptions of community development and urbanisation in relation to health and chronic disease risk. These methods enabled us to explore views on a range of environmental determinants, and also had the potential to reveal themes and ideas that may have previously been overlooked. In addition, we anticipated that the qualitative findings could be used to guide future community planning and interventions to reduce the growing burden of chronic disease in similar rural transitional communities.

We undertook a qualitative study between November 2013 and May 2014. The research was embedded within the Andhra Pradesh Children and Parents’ Study (APCAPS), a large intergenerational cohort study in 29 villages of rural India which looks at CVD risk in rapidly transitioning communities. APCAPS was established to follow up with households that participated in the Hyderabad Nutrition Trial (HNT), a population-based evaluation of the Integrated Child Development Services scheme, India's flagship community nutrition program that provides food, health, and education inputs to pregnant and lactating women and children under the age of 6 years (19). The HNT trial was carried out in 1987–1990 among 29 villages in two adjacent administrative areas approximately 50–100 km from the city of Hyderabad in southern India (20). The villages currently range in population size from 500–12,000 people. A complete description of the APCAPS cohort, including details of the initial HNT trial and all follow-up data collection, has been published previously (21).

### Study location and sample

Initially, we conducted three pilot FGDs across three APCAPS villages (number of participants=18, of which men=7, women=6, and teenage girls=5) in November 2013. Following the pilot, an additional six FGDs were conducted in two other APCAPS study villages. The villages were relatively large and urbanised (population size 2,000–6,000; maximum of 70% of people engaged in agriculture), and were purposively chosen to meet the study objectives. FGDs were conducted separately with men, women, and teenagers; teenage boys and girls were combined in one group because they are accustomed to sharing their views with each other at school and therefore did not need to be interviewed separately, whereas it was more culturally appropriate to interview male and female adults separately. Participants were not asked about, or selected according to, caste or religion because it is a culturally sensitive issue; however, each focus group was likely to be made of participants from similar backgrounds as these groups tend to cluster socially and geographically. Details of the study sample are provided in Table 1. The results from all nine focus groups were combined for this analysis.

**Table 1:** Study sample from nine focus group discussions (FGDs) in rural villages in South India

		Men^a^	Women^a^	Teenagers^a^
Pilot	N	7	6	5
	Age^b^	25–60 (38)	19–55 (35)	16–18 (17)
Village 1	N	7	7	5
	Age^b^	24–59 (42)	23–66 (39)	16–19 (17)
Village 2	N	8	6	6
	Age^b^	25–55 (43)	28–40 (37)	14–18 (16)
Total	N	22	19	16
	Average age	41	37	17

a Each cell represents one FGD

b Ages presented are ranges (average) in years.

### Data collection

Within each village, the field team purposively recruited participants. Members of the field team approached village leaders and asked them to identify possible subjects. The field team invited people from a range of ages and both sexes to take part to enable us to get a range of opinions on the topics of interest.

Questions for the pilot were based on *a priori* knowledge and observational evidence about chronic disease risk factors in the study villages. People were asked about their perceptions of the environment in which they lived, their knowledge of chronic disease risk, and how the two were linked. AH, SK, RJ, and AP used the results from the pilots to draft and review a more detailed topic guide in English. The field team translated the topic guide into Telugu and made some modifications to make it more relevant to the local context. Open and semi-structured questions were asked to prompt discussions of people's perceptions of the community environment and its effect on their health, focusing on key cardiovascular risk factors (diet, physical activity, tobacco use, and pollution), for example, ‘how do you think your village has changed in the last 10 years and how have these changes affected you?’ The main themes from the pilot were used to inform the final topic guide; questions which generated conversation were expanded, and those that did not were deemed not relevant and removed. Probes were used to clarify answers and obtain detailed information about particular topics raised in a FGD.

Two local fieldworkers from the National Institute of Nutrition (India) led the FGDs fully in Telugu and a third took notes. The FGDs were recorded using a voice recorder. Permission to record was requested verbally from all participants. The fieldworkers (who were all fluent in Telugu and English) translated the audio recordings, and transcribed them in English. Ten percent of the transcripts were checked by an additional native Telugu speaker to ensure accuracy and validity in translation and transcription; no major faults were found. For an additional assurance of the quality of the data once translated, the study team listened to the audio recording to ensure that the nuances of the language were captured accurately, and discussed any differences in opinion until a consensus was reached about the intended meaning. After the first two FGDs were conducted, the transcriptions were reviewed to see if the topic guide was adequately achieving the study objectives. Some small revisions to the language of the topic guide were made.

### Analysis

The data were analysed manually using the Framework approach; this involves five distinct but interconnected stages of analysis: familiarisation of the data; identifying and creating a thematic framework; indexing; charting and mapping; and finally interpretation (22). This method is designed specifically for use in applied research and is both a deductive and inductive process thereby enabling the research questions to be examined without precluding the emergence of new and unexpected findings. Initially, thematic codes were created deductively according to the questions asked (e.g. pollution). On indexing the data, further subthemes were identified inductively from the original thematic codes (e.g. air pollution and contamination within pollution). Results were summarised in Excel because the small number of transcripts did not require the use of qualitative data analysis software. A sample of codes was checked for quality assurance.

The study received ethical approval by the Public Health Foundation of India, the National Institute of Nutrition (India), and the London School of Hygiene and Tropical Medicine research ethics committees.

## Results

Nine FGDs were held across five villages in rural Andhra Pradesh, India. The FGDs lasted between 53 and 114 min. The total sample was 57 men, women, and teenage boys and girls (Table 1). All people who were approached took part. All the participants were from different households but many knew each other already. Each FGD was made of participants from similar socioeconomic backgrounds, caste, and religion in order to enhance people's willingness to talk and therefore no information on these factors is reported here.

The key issues discussed were sorted into four major themes of interest: the difference between urban and rural living and the effect of development on general health, diet, physical activity, and pollution. Each theme is reported below and elaborated further with illustrative quotes. Each quote is followed by the sex and age of the speaker, for example, (M, 15).

### A comparison of rural and urban living and the effect of development on general health

There were mixed opinions about whether the general health of rural or urban communities was better. Some participants thought that city dwellers were healthier because they had better access to medical facilities, were wealthier, and had better educational standards. Other participants thought that rural communities were healthier because of a cleaner environment, more opportunities for physical activity, increased exposure to the sun resulting in higher vitamin D levels, and the positive impact of greater social unity on mental health. Participants felt that in their villages there had been an increase in a number of chronic conditions in the past decade, including poor mental health (higher levels of stress, sleep disruption, and depression), more instances of cancer, more liver problems (attributable to increased alcohol consumption), and higher smoking rates. A number of men and women believed that there had been a reduction in life expectancy due to unhealthy lifestyles.

People said that they wanted more development in the villages to improve their health, particularly better medical facilities, educational institutions, and job opportunities: ‘when there is development, automatically we will be healthy. If all facilities are made available here then even nearby villagers would come to trade here. Then development would automatically occur and it would be very good for us’ (M, 35). The general consensus was that people wanted more development locally so that they had shorter journeys to reach essential services. Long journeys were cited by participants of all ages as a reason for poor health; women believed their children suffered as a result of needing to travel into Hyderabad for schooling, and men did not want to have to travel over 40 km to reach a medical facility. Although some participants, particularly those over 40 years old, said rural living conditions were better in the past, in general, development in villages was seen as a positive process: ‘40 years back the village conditions were very worse *[sic]* but now we have all facilities here and our life is good’ (F, 66).

Teenagers talked about inequity in development within their villages (‘the poor getting poorer and the rich getting richer’ (M, 16)), such that development and urbanisation had only benefited the rich and higher castes of the villages: ‘in the past, everyone was treated alike but now because of these changes people started showing indifferences … people treat those with big houses in one way and those without in another way’ (F, 16). This, they believed, had a negative impact on people's mental health: ‘when there is such discrimination in the community then poor people would go into depression and this would affect their health’ (M, 17).

### Diet

According to people in all FGDs, the food environment has changed. Reported changes in dietary habits included cooking less frequently and relying on food from outside, particularly eating more fast food: ‘back then we had nothing, but today everything is available. We didn't know what fast food was. Now, out of curiosity we go to have it’ (M, 24). People also reported a lack of patience and willingness to prepare food, for example, using traditional techniques such as hand grinding of flours: ‘development is stopping people from following good habits. I mean … in those days with whatever little machinery available people used to make food but now as so many mills and things have come up so did these changes in the food habits’ (F, 17).

Teenagers, in particular, were keen to see further changes in the dietary environment; for example, they wanted more bakeries and fast food outlets to be available in their villages. Changes in the consumption of fruits and vegetables were also discussed; there was now a greater variety available but people had to rely more on purchased items rather than those grown on their own farms. Some fruits were prohibitively expensive as they came from outside the village and ‘only the wealthy person will be able to afford and not bother about the price’ (M, 24). Food in cities was generally considered to be more expensive.

Many participants believed that people's dietary habits had changed for the worse, with a resultant increase in the prevalence of chronic conditions: ‘now if we eat fast food for about a week then we will definitely fall sick. But eating rice, dal and vegetables will always keep us healthy’ (M, 35) and: ‘I'm 62 years old now. People younger to me are suffering from diseases like knee pains, BP *[hypertension]*, leg pains and sugar *[diabetes]*. So, that means that there is a difference in food habits and living conditions, in those days and now’ (M, 62). Women also said that men tended to drink more alcohol than previously and described an inability to influence their husbands’ behaviour to help them reduce this increased alcohol consumption.

#### Quality of food

A key issue of concern to participants was the increased use of pesticides on crops: ‘we feel we are eating medicines instead of vegetables’ (F, 43). Food was perceived as less nutritious than in the past and men attributed this increased use of pesticides to poor health and reduced life expectancy: ‘the lifestyle *[in previous generations]* was very different and that's the reason old people are still healthy and living. People then were very strong. Now crops are grown with medicines, we can't follow the same lifestyle so we can't live for so long years as them *[sic]*’ (M, 35) and ‘in those days we had fresh air, no diseases were seen, but now *[all our food]* is adulterated’ (M, 59). Conversely, across all villages, a minority of people felt the availability of nutritious food was better than it had been previously.

### Physical activity

#### Occupation

One of the biggest perceived impacts of urbanisation on the community was a shift in occupation. People believed agriculture had gone down ‘drastically’; whereas the previous generation relied mainly on subsistence farming, many people were now working in non-manual jobs, for example, in shops and in ‘government jobs’. When asked how occupation affected people's health, one woman said ‘people are not that strong like they used to be in the past’ (F, 28); this was a common perception across all ages and both sexes. Generally, women (more so than men) believed that people in cities were less healthy because of the lack of ‘hard work we do in villages’, they are too sedentary, eat too much and ‘don't know what hardship is’ (F, 25). Physical activity for leisure or health was thought to be unnecessary as people believed occupational physical activity was sufficient.

Comparing themselves to city dwellers, one woman said: ‘*[people from Hyderabad]* are not healthy because they don't do hard work like us. We do so much of hard work till evening. They sit all day at home. They just eat and sit idle’ (F, 55). They were aware of the link between activity and obesity, saying that people in sedentary work were heavier, whereas ‘working in the fields is like exercise for us, we go to our fields and feel better’ (F, 36). Teenagers in particular were aware of the need to be active on a daily basis and that modern lifestyles in their village were preventing this: ‘people in offices don't do physical work, so, they are not healthy’ (M, 16), ‘no blood circulation occurs in people who sit and work’ (F, 17) and ‘office work causes digestive problems’ (M, 59). In contradiction to this, office workers were perceived to be more educated, ‘they know everything’ (F, 16) and had a better understanding of what food to eat, which teenagers believed enabled them to be healthier.

#### Open space

An interesting change associated with urbanisation, also affecting physical activity, was a reduction in open space, which ‘has reduced a lot and now we don't have anywhere to play’ (M, 15). This theme was widely discussed across all groups and participants said that the lack of facilities prevented people, particularly girls, women, and members of lower castes, from being physically active: ‘only Reddy people use the space … the others are not allowed’ (F, 16).

#### The sociocultural environment

The social environment in the study villages had a big impact on behaviour, physical activity, and therefore health, particularly for girls and women. Some of the men said that women were too scared to walk outside, that others may think badly of them if they did, and that ‘for ladies to walk on the roads is a matter of security’ (F, 28). With no women-only facilities available and family members discouraging them from being active, women were much less likely than men to take part in physical activity: ‘they say that grown up girls should not go out to play. They tell it to other people of the village and like *[that]* it spreads. People's mind-set should be changed’ (F, 15).

### Pollution

Pollution was cited as one of the worst aspects of urbanisation in relation to health. Increased levels of pollution (here referring to air pollution and water and soil contamination) were attributed to a number of sources, of which the most commonly cited were poultry farms and factories, traffic, open sewers and stagnant water, improper rubbish disposal, and pesticides. Generally, participants said levels of pollution were worse than 10 years ago (with the exception of water quality which had improved), but that there was still less pollution than in Hyderabad.

#### Air pollution

Across all of the FGDs, people reported an adverse effect of air pollution on their health. One woman said that rubbish gets dumped in open spaces and not removed promptly: ‘the stink *[makes]* our head pains, we feel giddy, suffer fever. Sometimes we faint because of that smell … People are suffering from different kinds of diseases, fever and also typhoid, malaria and jaundice’ (F, 35). One man said that the increase in pollution associated with development seriously affected everyone's health: ‘old people are suffering from joint pains. Now-a-days people of all age groups are suffering from joint pains. So small diseases like these and also cancer is seen now-a-days. In my opinion all these diseases are mainly caused due to the pollutants’ (M, 24).

Pollution from increased traffic had also significantly affected health in recent years; walkability had decreased because of ‘dangerous cars and lorries parked on the roads’ (M, 60). Teenagers said the traffic made them ‘feel scared’; it caused ‘headaches’ and ‘too much noise pollution’.

## Discussion

This qualitative study describes work undertaken in communities undergoing rapid transition in rural India. Within and between individuals, there were contradictory perceptions of the effect of urbanisation on health. Some of the changes associated with urbanisation were seen as positive (for example improved access to healthcare), but there was simultaneously nostalgia for the ‘old days’, during which time many participants believed their communities were healthier, less polluted, more physically active, with better access to nutritious food and longer life expectancies. Some issues particularly divided opinion, for example, food; teenagers felt that the greater dietary diversity available today was a positive change to be encouraged, but adults felt it was a reason for poor health. Residents viewed change and development within their villages as an inevitable and largely positive process but with negative health implications. They also believed that any benefits development would bring would not be equally distributed across all socioeconomic groups.

The health of rural communities in LMICs around the world has changed dramatically in recent decades as populations undergo industrialisation and become more integrated into global economies (23). Methods of agricultural production, employment, and food acquisition are shifting, with many populations switching from food to cash crop production, increasing their access to cash to purchase varied food items in markets, or migrating to urban centres looking for wage labour. As populations become increasingly integrated with Western markets and lifestyles, diets also shift (24). These changes have been documented in India, but at the national rather than local level (3).

Although there has been much speculation about possible associations between the social and built environment and health, empirical evidence is lacking, particularly in LMICs and in relatively rural areas. This study provides interesting data from a transitional community on perceptions of health as a result of the local environment. The people included in this study reported having experienced changes in diet, physical activity, occupation, and levels of pollution; all of these factors are known to contribute to chronic disease and the development of an ‘obesogenic’ environment (25–28). This population's chronic disease risk may rise in the coming years as urban influences increasingly permeate these communities, as evidence from urban areas would suggest (2–4).

Importantly, many participants described feelings which indicated a lack of empowerment to cope with the changes that were occurring. For example, there were strong feelings about the problem of pollution (factories and poultry farms encroaching on village boundaries and food producers using more pesticides) and concern about the associated health risks, but people felt they were unable to prevent these changes because of a lack of social unity to influence local authorities. Establishing community support groups and channels for advocacy may help communities to wield more influence with local policy makers.

There is a considerable amount of evidence indicating the impact that the sociocultural context and community environment has on levels of physical activity (7, 11, 12, 29, 30). This was similar within our sample; although there was a good understanding of the health benefits of being active, people believed that purposive physical activity was unnecessary because traditional lifestyles were sufficiently physically demanding. Conflictingly, people reported that physical activity levels had steadily reduced with an increase in sedentary occupations, but that they were not willing, or able, to respond individually to these changes (due to age, disability, and sociocultural restrictions). An individual's perception of their environment, including their ability to partake in physical activity within that environment, has a strong correlation with levels of activity (12, 31, 32). Self-empowerment and greater self-efficacy have successfully helped to change behaviour positively and reduce non-communicable disease risk in rural Indian communities (33). It is therefore important to reinforce messages of the benefits of physical activity across the life course, whilst simultaneously empowering communities to create facilities that enable them to be physically active. Behaviour change messages should be designed and delivered within the contextual determinants of health to be culturally acceptable and inclusive of all populations groups, to maximise the changes of mitigating chronic disease risk.

### Strengths and limitations

This study is one of the first from India to look at perceptions of the community environment with a focus on perceived changes over time. Although a number of surveys have attempted to capture the community environment and its impact on health over the past decade, most of these have been conducted in developed countries, or in purely urban areas, and therefore are not directly comparable to this study (34, 35). In addition, many have used objective measures to measure the community environment (36–38). The use of qualitative methods enabled a more ‘emic’ understanding of people's perceptions of their community environment; it also provided some data illustrating the perceived effects of rapid change and development on health.

Focus groups afford depth and insight into a research question and are particularly useful for providing rich, detailed data about people's perceptions on a certain matter (39). However, to ensure the data are reliable and to run a focus group successfully, a good moderator is essential; the moderator should be able to relate to the participants and be able to converse with them on an equal level. Knowledge of the local area helps moderators to develop a rapport with the participants and be able to relate to the issues participants are describing (40). The moderators were from the local area and already familiar with the study villages. In addition, all data were collected by the same moderators, increasing the reliability of the study findings. However, when analysis of qualitative data is conducted outside of a researcher's own linguistic or cultural community, there is a risk of misinterpreting the data and losing nuances. Although every effort was made to reduce this (validating a sample of the data with different translators and revisiting the original recordings to discuss the meaning of the data), some of the views of the participants may have been distorted.

This study was conducted in a small geographic area (all five villages are within the same district) and therefore care must be taken when extrapolating the findings. Including people from other villages at different stages of urbanisation, or from Hyderabad, may have provided more information. Additional resources would have allowed us to conduct individual interviews with key informants in the study villages, which may have enabled us to explore in more depth some of the issues raised by participants in the group discussions.

A number of the respondents were not accustomed to taking part in FGDs, and therefore may not have had the courage to speak. As is a common problem in FGDs, some people dominated the discussions, resulting in less confident members of the groups remaining silent, even after attempts by the moderators to include them. The FGDs with women provided much less data: women are generally not accustomed to sharing their opinions in public fora and were less able to express their opinions than the men in our sample, needing much more encouragement to speak; this suggests a lack of empowerment. They were acutely aware of this, stating that they were illiterate and therefore could not express their opinions. Spending more time with the women and building a rapport with them prior to the data collection may have provided an environment where the women felt more comfortable and generated a better discussion.

### Implications for further work

Understanding how changes in the community environment are affecting populations in transitional rural areas and how people relate to their environment is useful to guide community planning for health. Further research into this area, with larger sample sizes, is warranted and its results may be useful to design interventions aiming to reduce the burden of chronic disease. Including members from a range of social groups, for example, different socioeconomic strata, castes, and religions, may help target interventions where they are most needed and address the issue of inequitable development. Measures to educate and empower people to make healthy choices within their community may help reduce the spread of chronic disease risk factors in future years.

## Conclusions

In the present study, change and development within villages was viewed as an inevitable and largely positive process but not without negative health consequences. Although these villages remain relatively rural, they are increasingly exposed to outside influences. Understanding how these changes are affecting populations in rural areas and how people relate to their environment may be useful to guide community planning for health. Measures to educate and empower people to make healthy choices within their community may help reduce the spread of chronic disease risk factors in future years.

## Ethics

Ethical approval was sought from the necessary ethics committees at the Public Health Foundation of India, the National Institute of Nutrition (India), and the London School of Hygiene of Tropical Medicine, UK.
